# Dopamine Synthesis Capacity is Associated with D2/3 Receptor Binding but Not Dopamine Release

**DOI:** 10.1038/npp.2017.180

**Published:** 2017-10-04

**Authors:** Anne S Berry, Vyoma D Shah, Daniella J Furman, Robert L White III, Suzanne L Baker, James P O’Neil, Mustafa Janabi, Mark D’Esposito, William J Jagust

**Affiliations:** 1 Lawrence Berkeley National Laboratory, Berkeley, CA, USA; 2 Helen Wills Neuroscience Institute, University of California Berkeley, Berkeley, CA USA

## Abstract

Positron Emission Tomography (PET) imaging allows the estimation of multiple aspects of dopamine function including dopamine synthesis capacity, dopamine release, and D2/3 receptor binding. Though dopaminergic dysregulation characterizes a number of neuropsychiatric disorders including schizophrenia and addiction, there has been relatively little investigation into the nature of relationships across dopamine markers within healthy individuals. Here we used PET imaging in 40 healthy adults to compare, within individuals, the estimates of dopamine synthesis capacity (K_i_) using 6-[^18^F]fluoro-l-m-tyrosine ([^18^F]FMT; a substrate for aromatic amino acid decarboxylase), baseline D2/3 receptor-binding potential using [^11^C]raclopride (a weak competitive D2/3 receptor antagonist), and dopamine release using [^11^C]raclopride paired with oral methylphenidate administration. Methylphenidate increases synaptic dopamine by blocking the dopamine transporter. We estimated dopamine release by contrasting baseline D2/3 receptor binding and D2/3 receptor binding following methylphenidate. Analysis of relationships among the three measurements within striatal regions of interest revealed a positive correlation between [^18^F]FMT K_i_ and the baseline (placebo) [^11^C]raclopride measure, such that participants with greater synthesis capacity showed higher D2/3 receptor-binding potential. In contrast, there was no relationship between [^18^F]FMT and methylphenidate-induced [^11^C]raclopride displacement. These findings shed light on the nature of regulation between pre- and postsynaptic dopamine function in healthy adults, which may serve as a template from which to identify and describe alteration with disease.

## Introduction

In humans, *in vivo* PET imaging can be used to assess the function of multiple components of the dopamine system by targeting presynaptic markers of synthesis and transport, and postsynaptic markers of receptor-binding potential.

Given the highly regulated nature of the dopamine system, it is possible that pre- and postsynaptic function is dynamically adjusted to achieve a stable homeostatic balance in healthy adults. However, the nature of these relationships is not well understood, as multiple components of the dopamine system are rarely studied within the same individuals (though see Lee *et al*, 2000; Nandhagopal *et al*, 2009; Nandhagopal *et al*, 2011 for examples of multi-tracer studies of presynaptic function in Parkinson's disease; and Ito *et al*, 2017 for example in healthy adults). In this study, we aimed to characterize the relationship between dopamine synthesis capacity using the PET radioligand 6-[^18^F]fluoro-l-m-tyrosine ([^18^F]FMT), baseline D2/3 receptor-binding potential using [^11^C]raclopride, and dopamine release using methylphenidate-paired [^11^C]raclopride PET in healthy adults.

There has been little rigorous investigation into the association between PET markers of dopamine synthesis and receptor binding, though previous studies have begun to address this question with inconsistent results. A single study reported a negative relationship between dopamine synthesis capacity measured using [β-^11^C]dihydroxyphenylalanine (DOPA) and baseline D2/3 receptor binding measured using [^11^C]raclopride (Ito *et al*, 2011), and two studies have reported no relationship between dopamine synthesis capacity measured using [^18^F]DOPA and baseline D2/3 receptor binding measured using [^18^F]desmethoxyfallypride (Heinz *et al*, 2005; Kienast *et al*, 2008). This study design represents a departure from these previous investigations by (1) using a substantially larger sample size (*n*=40 compared to *n*=12–14) (2) measuring dopamine synthesis capacity using [^18^F]FMT, which has an improved signal to noise ratio relative to [^11^C and ^18^F]DOPA ligands (Sossi *et al*, 2002) and (3) relating dopamine synthesis capacity to dopamine release in addition to baseline D2/3 receptor-binding potential, where signal is influenced both by the density and avidity of receptors as well as by the concentration of synaptic dopamine.

To measure individual differences in dopamine release, we compared baseline [^11^C]raclopride signal (non-displaceable binding potential (BP_ND_)) with [^11^C]raclopride BP_ND_ following methylphenidate administration. There is a rich history of research establishing effects of pharmacological challenge on PET and SPECT measures of the dopamine system. For example, early studies established that treatment with amphetamine, which stimulates dopamine release (Kuczenski and Segal, 1989; Sharp *et al*, 1987), also reduces radioligand signal for tracers that bind to dopamine receptors (Breier *et al*, 1997; Kohler *et al*, 1981; Laruelle *et al*, 1996; Laruelle *et al*, 1995; Laruelle *et al*, 1997; Martinez *et al*, 2003; Ross and Jackson, 1989a; Ross *et al*, 1989b; Young *et al*, 1991). Released dopamine accumulates in the synapse and competes with the radioligand for postsynaptic receptor binding, thus causing reduction in signal following amphetamine treatment. Methods for estimating *in vivo* changes in extracellular dopamine concentration using PET and SPECT imaging methods have been validated in elegant studies pairing microdialysis and imaging in nonhuman primates following amphetamine exposure (Breier *et al*, 1997; Endres *et al*, 1997; Laruelle *et al*, 1997; Narendran *et al*, 2014; Tsukada *et al*, 1999). These studies confirmed negative correlations between measured increases in extracellular dopamine concentration and reduced radioligand binding.

Building from findings using amphetamine, studies pairing methylphenidate administration with [^11^C]raclopride have established effects of unstimulated, endogenous dopamine release on D2/3 receptor binding. Methylphenidate does not stimulate dopamine release, but increases synaptic concentrations of dopamine by reducing dopamine reuptake via dopamine transporter blockade (Kuczenski and Segal, 1997; Volkow *et al*, 1998b). In a series of studies in humans, Volkow *et al* (1999) demonstrated that intravenous and oral methylphenidate (Volkow *et al*, 2001; Volkow *et al*, 2002) significantly reduced [^11^C]raclopride BP_ND_ in striatum, consistent with findings for amphetamine-stimulated release. Next, Volkow *et al* (2002) examined whether individual differences in the extent of methylphendiate’s blockade of the dopamine transporter explained observed variability in the magnitude of [^11^C]raclopride signal reduction. Change in dopamine transporter binding following oral methylphenidate was measured with [^11^C]cocaine and compared to change in [^11^C]raclopride binding. There was no significant relationship between these measures, which has been interpreted to indicate that individual differences in [^11^C]raclopride binding arise from differences in the activity of dopamine-releasing neurons rather than differences in transporter blockade. Therefore, individuals with low dopaminergic neuronal activity would have smaller increases in synaptic dopamine concentrations relative to individuals with high activity. To date, it is not known whether higher dopamine cell activity and release are associated with increased dopamine synthesis capacity.

This study comprehensively compared measures of [^18^F]FMT K_i_, baseline [^11^C]raclopride BP_ND_, and dopamine release within subjects with the aim of addressing the fundamental question of how pre- and postsynaptic components of the dopamine system are interrelated. This study significantly advances efforts in the field to understand basic dopaminergic function in humans by empirically testing multi-tracer relationships that have not, to our knowledge, been investigated previously. We hypothesized that higher levels of dopamine release, putatively reflecting higher dopaminergic neuronal activity, would positively predict individual differences in dopamine synthesis capacity in healthy young participants. We did not have a strong hypothesis regarding the nature of relationships between dopamine synthesis capacity and baseline D2/3 receptor binding given the mixed evidence to date (Heinz *et al*, 2005; Ito *et al*, 2011; Kienast *et al*, 2008).

## Materials and methods

### Participants

40 participants (18–25 years old, Mean=21.33, SD=1.99; men/women=15/25; 23 Asian, 7 Hispanic or Latino, 6 White (not Hispanic or Latino), 2 Black or African-American, 2 more than one race) underwent PET and MRI scanning. Power analyses determined that this sample size is sufficient to detect relationships of *r*=0.32 with achieved power of 0.80. Power analyses were conducted with G*Power 3.1.7 (Faul *et al*, 2007). The Institutional Review Boards at the University of California, Berkeley and Lawrence Berkeley National Laboratory approved the study. All participants provided written consent and received monetary compensation for participating in the study.

Participants were recruited as part of a larger ongoing study of dopaminergic mechanisms of cognitive control, which included three fMRI sessions, and self-report questionnaires. Analysis of fMRI results, and self-report measures is ongoing, and is not presented in the current report. Prior to enrollment, participants underwent medical screening and physical examination by a medical doctor or nurse practitioner. Participants did not have a history of neurological, psychological, or psychiatric disorder. Four participants reported having seen a psychiatrist or psychologist to treat school or family stress that was resolved at the time of enrollment, and did not require pharmacological treatment. Participants reported no symptoms of depression, anxiety, paranoia or hallucinations, homicidal thoughts or acts, violent or threatening behavior, suicidal thoughts or acts, or suicide attempts. Self-report measures were collected through paper and pencil questionnaires.

Exclusion criteria included consumption of more than 7 alcoholic drinks per week, and use of psychoactive drugs within 2 weeks of enrollment or 10 times in the past year. We assessed drug history using a written screening form. Participants indicated their drug use history for the following list of specific drugs as well as broader drug categories: cocaine, stimulants (other than caffeine), amphetamines, hallucinogens, ‘ecstasy’, opiates, sedatives, pain or sleeping pills, and marijuana. In addition, we tested drug and alcohol use via urine drug screening and alcohol breath test prior to enrollment. No participant tested positive for any psychoactive drug, and alcohol breath test confirmed alcohol concentration below 0.05%. Prior to the PET and MRI sessions, participants underwent additional screening for self-reported drug use including screening for methylphenidate, dexmethylphenidate, dextroamphetamine, lisdexamfetamine, amphetamine and methamphetamine. Reported medications were limited to birth control, antibiotics, asthma and allergy medication, and non-prescription pain relievers. Participants did not use nicotine with the exception of two participants who reported smoking 1–2 cigarettes per week. Exclusion of these two participants does not change the significance of our analyses (data not shown).

### Structural MRI Scan

Images were acquired using a Siemens 3 T Trio Tim scanner with a 12-channel coil. Each participant was scanned 3 times using a high-resolution T1-weighted magnetization prepared rapid gradient echo (MPRAGE) whole brain scan (TR=2,300 ms; TE=2.98 ms; FA=9**°**; matrix=240 × 256; FOV=256; sagittal plane; voxel size=1 × 1 × 1 mm; 160 slices). MPRAGE scans were aligned, averaged and segmented using FreeSurfer version 5.1 (http://surfer.nmr.mgh.harvard.edu/) and were used for coregistration with the PET data. The 3 MPRAGE scans were averaged to minimize the effect of head motion on the quality of image segmentation.

### [^18^F]FMT PET Data Acquisition

Participants underwent an [^18^F]FMT PET scan to measure dopamine synthesis capacity. [^18^F]FMT is similar to DOPA ligands as both tracers are substrates for aromatic amino acid decarboxylase, an enzyme in the dopamine synthesis pathway. Though not the rate-limiting step, its activity provides an estimate of dopamine synthesis capacity when provided with enough substrate (DeJesus, 2003). [^18^F]FMT does not undergo post-release processing as DOPA ligands do, but is instead trapped in the presynaptic terminal after its conversion to 6-[^18^F]fluorohydroxyphenylacetic acid (Jordan *et al*, 1997). Furthermore, it is not subject to methylation by catechol-O-methyltransferase as DOPA ligands are, with the consequence that radiolabeled metabolites do not enter the brain. Both of these factors result in improved signal to noise ratio in [^18^F]FMT images compared to DOPA ligands.

[^18^F]FMT was synthesized at Lawrence Berkeley National Laboratory using methods previously described (VanBrocklin *et al*, 2004). Participants ingested 2.5 mg/kg of carbidopa ~1 h before scanning to minimize the peripheral decarboxylation of [^18^F]FMT (Boyes *et al*, 1986; Firnau *et al*, 1988; Hoffman *et al*, 1992; Melega *et al*, 1990). All PET data were acquired using a Siemens Biograph Truepoint 6 PET/CT scanner (Siemens Medical Systems, Erlangen, Germany). After a short CT scan, participants were injected with approximately 2.5 mCi of [^18^F]FMT as a bolus in an antecubital vein (M±SD; specific activity=947.30±140.26 mCi/mmol; dose=2.43±0.06 mCi). Dynamic acquisition frames were obtained over 90 min in 3D mode (25 frames total: 5 × 1, 3 × 2, 3 × 3, 14 × 5 min). Data were reconstructed using an ordered subset expectation maximization algorithm with weighted attenuation, corrected for scatter, and smoothed with a 4 mm full width at half maximum (FWHM) kernel.

### [^11^C]raclopride PET Data Acquisition

Participants received two [^11^C]raclopride PET scans an average of 21.65 days before or after the [^18^F]FMT scan (median=7 days) to measure D2/3 receptor occupancy and dopamine release. [^11^C]raclopride is a D2/3 receptor antagonist with relatively low affinity (Kd=1.2 nM) that competes with endogenous dopamine (Kohler *et al*, 1985; Seeman *et al*, 1989). [^11^C]raclopride was synthesized at Lawrence Berkeley National Laboratory using methods previously described (Volkow *et al*, 1993). To measure baseline D2/3 receptor occupancy, participants ingested a placebo pill approximately 1 h before [^11^C]raclopride scan 1. The placebo scan was always performed first. To measure dopamine release, participants ingested 30 mg (M±SD mg/kg: 0.46±0.08) of methylphenidate ~1 h before [^11^C]raclopride scan 2. Endogenous dopamine release was measured as the percent change in BP_ND_ from [^11^C]raclopride scan 1 to [^11^C]raclopride scan 2 ((placebo [^11^C]raclopride−methylphenidate [^11^C]raclopride)/placebo [^11^C]raclopride). Scans were conducted on the same day, 2 h apart and participants were blind to whether placebo or methylphenidate was administered. The 30 mg pill provides a smaller dose than the 60 mg pill used in previous studies (Broft *et al*, 2012; Clatworthy *et al*, 2009; Martinez *et al*, 2011; Martinez *et al*, 2012; Volkow *et al*, 2001; Volkow *et al*, 2002). The fixed mg amount used here and by others has the disadvantage of not accounting for individual differences in body weight. Our pilot testing determined the 30 mg pill produced a percent reduction in [^11^C]raclopride signal within the range of signal reduction in [^11^C]raclopride BP_ND_ associated with cognitive task performance: 5.3–10.2% (Jonasson *et al*, 2014; Monchi *et al*, 2006). For both [^11^C]raclopride scan 1 and [^11^C]raclopride scan 2, after a short CT scan, participants were injected with approximately 10 mCi of [^11^C]raclopride as a bolus in an antecubital vein. Mean specific activity and dose were not significantly different for [^11^C]raclopride scan 1 (M±SD; specific activity=5280.45±1359.41 Ci/mmol, dose=9.83±0.07 mCi) and [^11^C]raclopride scan 2 (specific activity=5092.98±1533.82 Ci/mmol, dose=9.83±0.09 mCi) as assessed by paired *t*-tests (specific activity *t*(39)=1.08, *p*=0.29, dz=0.17; dose *t*(39)=0.27, *p*=0.79, dz=0.00). Dynamic acquisition frames were obtained over 60 min in 3D mode (19 frames total: 5 × 1, 3 × 2, 3 × 3, 8 × 5). Reconstruction was performed as described above.

### PET Data Analysis

PET data were preprocessed using SPM8 software (Friston *et al*, 2007). To correct for motion between frames, images were realigned to the middle frame. The first five images were summed prior to realignment to improve realignment accuracy, as these early images have relatively low signal contrast. Structural images were coregistered to PET images using the mean image of frames corresponding to the first 20 min of acquisition as a target. The mean image for the first 20 min was used rather than the mean image for the whole scan time because it provides a greater range in image contrast outside of striatum thus making it a better target for coregistration.

**Figure 1:**
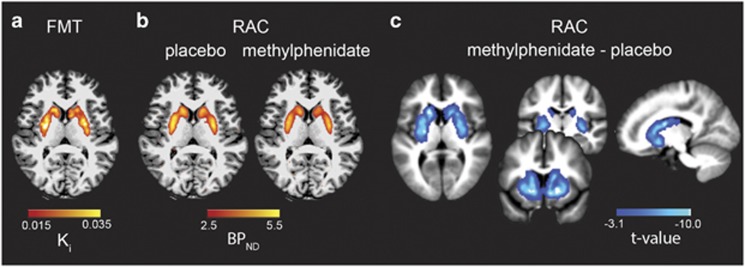
Within-subject measures of dopamine synthesis capacity and D2/3 receptor binding. (a) [^18^F]FMT K_i_ signal reflecting dopamine synthesis capacity was measured throughout striatum. The axial slice illustrates the extent of the striatal K_i_ signal for a representative subject overlaid on the subject’s native space T1 MPRAGE. (b) [^11^C]raclopride BP_ND_ displayed for placebo (baseline) scan as well as post-methylphenidate scan for the same representative subject. Methylphenidate administration reduced [^11^C]raclopride BP_ND._ (c) Striatal regions showing significantly reduced [^11^C]raclopride BP_ND_ following methylphenidate administration across all participants. The t-map for the paired t-test comparing baseline and post-methylphenidate [^11^C]raclopride BP_ND_ is displayed on the normalized mean T1 MPRAGE for all subjects.

For [^18^F]FMT PET, graphical analysis for irreversible tracer binding was performed using Patlak plotting (Patlak and Blasberg, 1985; Sossi *et al*, 2003) implemented using in-house software and Matlab version 8.2 (The MathWorks, Natick, MA). Without measurement of the arterial input function, both [^18^F]FMT and [^11^C]raclopride PET analysis used reference region models. Such analyses rely on the existence of a tissue region with few specific binding sites (Blomqvist *et al*, 1989; Cunningham *et al*, 1991). Cerebellar gray matter was used as the reference region because this region shows very little tracer uptake, and has an extremely low density of dopamine receptors and metabolites relative to striatum (Camps *et al*, 1989; Farde *et al*, 1986; Hall *et al*, 1994; Levey *et al*, 1993). The most anterior ¼ of cerebellar gray was removed from the reference region to limit contamination of signal from the substantia nigra and ventral tegmental area. Exclusion of the anterior portion of the cerebellar gray has been reported previously (Aarts *et al*, 2014; Berry *et al*, 2016; Braskie *et al*, 2011; Braskie *et al*, 2008; Dang *et al*, 2017; Dang *et al*, 2012a; Dang *et al*, 2012b, 2013; Dang *et al*, 2016; Klostermann *et al*, 2012; Smith *et al*, 2016; Wallace *et al*, 2014), and was performed by manually removing the anterior ¼ of coronal slices from individual participants’ native space cerebellar gray FreeSurfer segmentation using Mango software (http://ric.uthscsa.edu/mango/). K_i_ images were generated from PET frames corresponding to 25 to 90 min (Ito *et al*, 2006; Ito *et al*, 2007), which represent the amount of tracer accumulated in the brain relative to the reference region. K_i_ can be expressed as *K*_i_=*k*_2_*k*_3_/(*k*_2_+*k*_3_), where *k*_2_ is the rate constant for the return of free [^18^F]FMT from brain back to plasma and *k*_3_ is the rate constant for the trapping of brain [^18^F]FMT by aromatic amino acid decarboxylase. These images are comparable to *K*_i_ images obtained using a blood input function but are scaled to the volume of tracer distribution in the reference region (Figure 1a).

For [^11^C]raclopride PET, reversible tracer binding was quantified using simplified reference tissue model analysis (SRTM; Lammertsma and Hume, 1996). Specifically, a basis function version of the SRTM was applied as previously described (Gunn *et al*, 1997) with posterior cerebellar gray matter used as the reference region. Using this method, the time-activity curve of the brain region of interest is described relative to the reference region. This analysis assumes the reference region has no specific binding and that both regions have the same level of nondisplaceable binding (Gunn *et al*, 1997; Lammertsma and Hume, 1996; Salinas *et al*, 2015). The SRTM analysis was performed using in-house software provided by Dr Roger Gunn and Matlab version 8.2. SRTM analysis was used to determine BP_ND_, which can be defined as:

BP_ND_= f_ND_ × B_avail_/K_D_

where *B*_avail_ is the concentration of D2/3 receptors, *K*_D_ is the inverse of the affinity of the radiotracer for D2/3 receptors, and f_ND_ is the free fraction of the ligand in the nondisplaceable tissue compartment (Innis *et al*, 2007; Slifstein and Laruelle, 2001). A BP_ND_ voxel-wise map was generated for each participant (Figures 1b and c).

The use of BP_ND_ relies on the assumption that nondispaceable binding is independent of treatment effects. Methylphenidate administration has been shown not to alter cerebellar [^11^C]raclopride signal following 60 mg oral administration (Volkow *et al*, 2001; Volkow *et al*, 2002). It is possible that intravenous methylphenidate administration reduces cerebellar distribution volume (Volkow *et al*, 2014), though these results are not consistent (Volkow *et al*, 1999). Without measurement of the arterial input function, we could not directly test the effect of 30 mg oral administration cerebellar BP. We did, however, confirm that the cerebellar region of interest (ROI) did not show significant changes in BP_ND_ between [^11^C]raclopride scans 1 and 2 when using occipital cortex as the reference region (*t*(39)=0.70, *p*=0.49, dz=0.11). Occipital cortex also did not show significant changes in BP_ND_ between [^11^C]raclopride scans 1 and 2 when posterior cerebellar gray was used as the reference region (*t*(39)=0.47, *p*=0.64, dz=0.07).

### Regions of Interest

An ROI approach was used to test relationships between [^18^F]FMT K_i_, baseline [^11^C]raclopride BP_ND_, and percent change in [^11^C]raclopride BP_ND_ (dopamine release). ROI analyses were conducted in two ways. First, a single striatal ROI mask (henceforth referred to as ‘whole striatum’) was generated from group level voxel-wise analyses of K_i_ and BP_ND_ maps. K_i_ and BP_ND_ maps were spatially normalized to the TPM.nii template in MNI space, and smoothed with a 4 mm FWHM kernel in SPM 12. Two one-sample t-tests were performed to define significant voxels for [^18^F]FMT K_i_ and baseline [^11^C]raclopride BP_ND_. Paired *t*-test determined voxels for which methylphenidate significantly reduced BP_ND_. An initial cluster forming threshold of *p*<0.001 was applied. An additional minimum cluster extent threshold (*k*=55, *p* <0.05) was applied using 3dClustSim in AFNI (https://afni.nimh.nih.gov/). The whole striatum mask was comprised of the intersection of voxels (7097 mm^3^) surviving group level testing for [^18^F]FMT K_i_ and baseline [^11^C]raclopride BP_ND_ one-sample *t*-tests, and change in [^11^C]raclopride BP_ND_ paired *t*-test ([^18^F]FMT K_i_∩baseline [^11^C]raclopride BP_ND_∩placebo [^11^C]raclopride BP_ND_>methyphenidate [^11^C]raclopride BP_ND_).

Secondary, exploratory analyses examined the consistency of relationships between [^18^F]FMT K_i_ and [^11^C]raclopride BP_ND_ measures in striatal subregions. Striatal subregions were manually drawn for each participant. ROIs were drawn in native space on each participant’s averaged MPRAGE MRI scan using Mango software. The dorsal caudate, dorsal putamen, and ventral striatum were drawn as previously described (Mawlawi *et al*, 2001). This manual segmentation protocol was designed to create structurally defined ROIs that reflect the dorsal–ventral functional organization of the striatum. Specifically, ventral aspects of caudate and putamen are included in the ventral striatum ROI along with nucleus accumbens. These ventral portions of caudate and putamen partially surround nucleus accumbens, and share cortical and subcortical inputs from the limbic system (Haber *et al*, 1994; Poletti and Creswell, 1977; Russchen *et al*, 1985; Van Hoesen *et al*, 1981; Yeterian and Van Hoesen, 1978). Inter-rater reliability was high for manually drawn striatal subregions. For ROIs of five participants drawn by 3 raters, the Sorensen-Dice coefficient ranged from 0.80 to 0.89, and the intra-class correlation coefficient ranged from 0.87 to 0.99 for PET [^18^F]FMT K_i_ signal extracted from ROIs. Mean±SD ROI volumes were 2042±377 mm^3^ for dorsal caudate, 3759±608 mm^3^ for dorsal putamen, and 1788±330 mm^3^ for ventral striatum.

### Statistical Analyses

Statistical analyses were performed using SPSS, version 24. Analyses compared [^18^F]FMT and [^11^C]raclopride measures in striatum. Pearson correlations tested relationships between striatal [^18^F]FMT K_i_, [^11^C]raclopride BP_ND_, and dopamine release (% change: ([^11^C]raclopride BP_ND_ placebo−[^11^C]raclopride BP_ND_ methylphenidate)/[^11^C]raclopride BP_ND_ placebo). Primary correlation analyses were performed for whole striatum defined in MNI space. Secondary analyses tested correlations within striatal subregions (dorsal caudate nucleus, dorsal putamen, ventral striatum) as described above.

Shapiro-Wilk tests confirmed distributions were normal for PET signal in all regions with the exception of percent change in [^11^C]raclopride BP_ND_ for whole striatum; a Spearman correlation is reported for the analysis of its relationship with [^18^F]FMT K_i_. Correlations between [^18^F]FMT and [^11^C]raclopride % change are corrected for individual differences in body weight. We report *r* and *p*-values along with 95% confidence intervals for the *r*-values based on 1000 bootstrap samples (*r*, (confidence interval), *p*).

Complementary analyses demonstrated the limited impact of partial volume effects on our results. For analyses on manually drawn striatal subregions, we confirmed that all correlations described above remained significant after covarying ROI volume. Second, we confirmed that applying ROI-based partial volume correction (PVC; Rousset *et al*, 1998) to PET data did not affect our main conclusions. These analyses were performed in native space (non-normalized data) and correct for between-subject differences in the inclusion of white matter and CSF in the measured volumes. To apply the PVC in native space, we used FreeSurfer-generated ROIs for gray matter cortical and subcortical regions, white matter, and cerebral spinal fluid with manually drawn striatal ROIs substituting for the automated striatal segmentation. PVC results are reported in Supplementary information.

## Results

### Reduction of [^11^C]raclopride BP_ND_ Post Methylphenidate

Overall, [^11^C]raclopride BP_ND_ decreased 8.78±4.23% post-methylphenidate (M±SD for whole striatum). The effect of methylphenidate on [^11^C]raclopride BP_ND_ is visualized in the voxel-wise paired t-test comparing BP_ND_ following placebo *vs* BP_ND_ following methylphenidate (Figure 1c). A single cluster comprised the entire striatum (peak: MNI 18, 16, −6, *k*=7097). The location of the peak in ventral striatum is consistent with previous reports (Drevets *et al*, 1999). [^18^F]FMT K_i_, [^11^C]raclopride BP_ND_, and percent change in [^11^C]raclopride BP_ND_ values for whole striatum and striatal subregion ROIs are reported in Table 1. PVC values are reported in Supplementary Table S1.

### Relationship Between Striatal [^18^F]FMT K_i_ and [^11^C]raclopride BP_ND_

**Figure 2:**
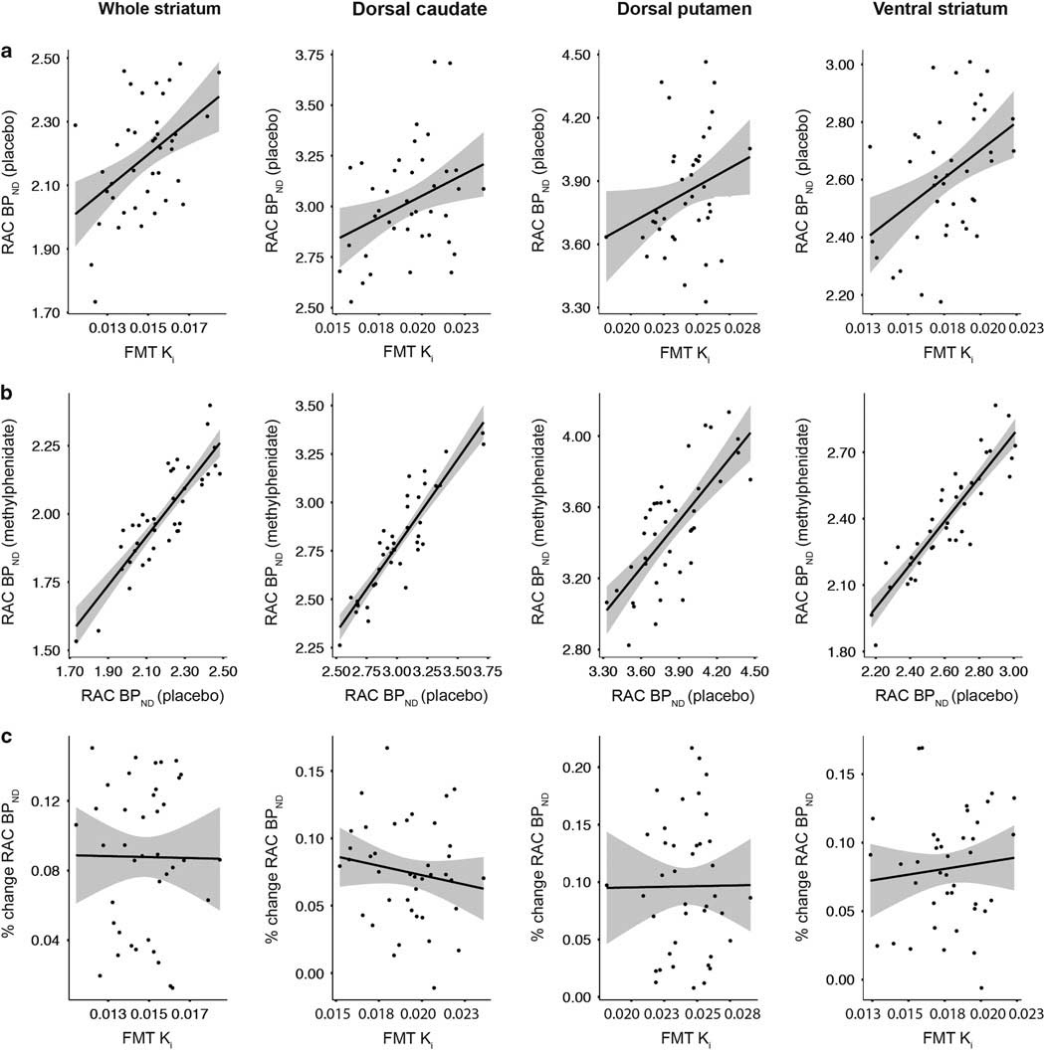
Relationships between [^18^F]FMT K_i_ and [^11^C]raclopride BP_ND_ for striatal regions of interest. (a) Baseline [^11^C]raclopride BP_ND_ following placebo was positively related to [^18^F]FMT K_i_ the whole striatum region of interest (ROI) derived from voxel-wise analyses (*r*=0.46, *p*=0.003). Baseline [^11^C]raclopride BP_ND_ and [^18^F]FMT K_i_ were positively related for manually drawn ROIs in dorsal caudate (*r*=0.34, *p*=0.03) and ventral striatum (*r*=0.41, *p*=0.008), and related in dorsal putamen at trend level (*r*=0.26, *p*=0.10). (b) [^11^C]raclopride BP_ND_ following methylphenidate and placebo (baseline) were highly correlated (*r*=0.72–0.90, all *p*<0.0001). (c) Dopamine release (([^11^C]raclopride placebo-[^11^C]raclopride methylphenidate)/ [^11^C]raclopride placebo) was not related to [^18^F]FMT K_i_ (*r*=−0.15–0.11, all *p*>0.35). [^11^C]raclopride is abbreviated as RAC.

Pearson correlations showed significant positive relationships between [^18^F]FMT K_i_ and baseline [^11^C]raclopride BP_ND_ for whole striatum (*r*=0.46 [0.17, 0.70], *p*=0.003). This relationship was generally consistent across striatal subregions, as there were positive correlations in dorsal caudate and ventral striatum, and a relationship in dorsal putamen at trend level (Figure 2a; Table 2). Controlling for region volume did not change the reported r-values (data not shown). Further, positive relationships remained following PVC (Supplementary Table S2).

Pearson correlations showed strong positive relationships between baseline [^11^C]raclopride BP_ND_ and post-methylphenidate [^11^C]raclopride BP_ND_ for the whole striatum ROI (*r*=0.86 [0.76, 0.92], *p*<0.001). Considering striatal subregions independently, there were positive relationships for all ROIs (Figure 2b; Table 2). Controlling for region volume did not change the statistical significance of any relationship, though reduced the *r*-value for ventral striatum by 0.01 (data not shown).

There were no correlations between body weight and individual differences in dopamine release (percent change in [^11^C]raclopride BP_ND_ after methylphenidate) in striatal ROIs for the fixed 30 mg methylphenidate amount (Spearman’s *r*=0.04–0.11, all *p*>0.49). Spearman correlations were used as Shapiro-Wilks test indicated body weights were not normally distributed (*W*=0.91, *p*=0.004).

We did not find a relationship between [^18^F]FMT K_i_ and dopamine release for whole striatum (*r*=−0.01 (−0.31, 0.33), *p*=0.97; for individual ROI results see Figure 2c, Table 2). Controlling for region volume did not change the reported r-values (data not shown). As a further test of the relationship between [^18^F]FMT and dopamine release, data from each striatal ROI were submitted to separate multiple regression models with post-methylphenidate [^11^C]raclopride BP_ND_ as the dependent variable, and predictors [^18^F]FMT K_i_, baseline [^11^C]raclopride BP_ND_, and region volume. Region volume was not included in the model for the whole striatum ROI, which was defined from normalized group voxel-wise maps and was therefore consistent across subjects. Models for all striatal regions were significant (all F(3,36)>13.22, all *p*<5.67 × 10^−6^, adjusted *r*^2^=0.48–0.80). Baseline [^11^C]raclopride BP_ND_ significantly predicted post-methylphenidate [^11^C]raclopride BP_ND_ for all models (all *t*>5.99, *p*<7.18 × 10^−7^), and [^18^F]FMT K_i_ did not predict post-methylphenidate [^11^C]raclopride BP_ND_ for any model (all *t*<0.99, *p*>0.33).

## Discussion

This study examined relationships between dopamine PET measures of striatal synthesis capacity, baseline striatal D2/3 receptor binding, and striatal dopamine release in a sample of 40 healthy young adults. We found a positive relationship between the presynaptic measure of dopamine synthesis capacity and the postsynaptic measure of baseline D2/3 binding. However, relationships between dopamine synthesis capacity and dopamine release were not evident. Below we describe the major implications of these findings and their caveats.

There are few studies that have investigated the relationships among dopamine PET measures within individuals. To our knowledge, there are only three other studies that have examined the relationship between presynaptic dopamine synthesis capacity and unstimulated D2/3 receptor binding using PET. Though two reported no relationship (Heinz *et al*, 2005; Kienast *et al*, 2008), it is possible these studies were underpowered. The Heinz study included 13 healthy controls and 12 alcoholic patients and the Kienast study included 12 healthy controls. Power analyses of the current study’s correlation strengths indicated at least 32 subjects were required to measure the relationship between [^18^F]FMT K_i_ and baseline [^11^C]raclopride BP_ND_ in whole striatum with power of 0.80 (G*Power; Faul *et al*, 2007). Ito *et al* (2011) reported a negative relationship between synthesis capacity (estimated using [^11^C]DOPA PET) and D2/3 receptor binding (estimated using [^11^C]raclopride PET) for the average of signal measured in caudate and putamen. The authors interpreted these findings to suggest either a compensatory relationship between pre- and postsynaptic dopamine function, or the effects of greater synaptic dopamine competing with [^11^C]raclopride for receptor binding in individuals with higher dopamine synthesis. A compensatory response could be mediated by lower D2/3 autoreceptor function, which has been linked to regulation of activity, but not *de novo* synthesis of aromatic amino acid decarboxylase (Cho *et al*, 1999; Zhu *et al*, 1992).

In contrast to the Ito study, we found positive rather than negative relationships between dopamine synthesis capacity and baseline D2/3 receptor binding. Critically, partial volume effects could not account for these correlations. Statistically controlling for ROI volume and formal PVC did not eliminate these positive relationships. The use of [^18^F]FMT as opposed to [^11^C]DOPA for estimating dopamine synthesis capacity likely represents the major source of discrepancy between studies. While [^18^F]FMT and DOPA ligands both act as substrates for aromatic amino acid decarboxylase, DOPA ligands are subject to additional *in vivo* metabolism not specific to the dopamine synthesis cascade including transport into vesicles and post-release processing at longer scan times (Sossi *et al*, 2002). This release and metabolism complicates the interpretation of DOPA ligands’ signal, which has been suggested to reflect dopamine turnover rather than synthesis capacity (Dejesus *et al*, 2001). In contrast, [^18^F]FMT is trapped in the presynaptic terminal following its conversion to fluoro-m-hyroxyphenylacetic acid (Jordan *et al*, 1997). In cases in which both tracers have been measured within subject, the [^18^F]DOPA tracer’s estimation of turnover rather than synthesis capacity has been implicated in the inversion of relationships observed for [^18^F]FMT (Dejesus *et al*, 2001). Therefore, it is possible that the negative relationship between [^11^C]DOPA and D2/3 receptor binding reported by Ito *et al* (2011) is driven by poorer estimates of dopamine synthesis capacity, or captures an inverse relationship between dopamine turnover (release and metabolism) and D2/3 receptor binding.

One question to consider is what are the functional and structural drivers underlying the positive relationship we observed between dopamine synthesis capacity and D2/3 receptor binding. During development, the number of dopamine-producing neurons innervating the striatum may affect the structural development and arborization of dendrites (McAllister, 2000; Whitford *et al*, 2002). Hence, the underlying structure (ie, number of synapses and the dendritic branching) may produce positive relationships between pre- and postsynaptic dopamine measures across subjects. Functional studies in animal models indicate that changes in afferent stimulation continue to shape postsynaptic structure (Ingham *et al*, 1989; Robinson *et al*, 2001; Robinson and Kolb, 1997; Wang and Deutch, 2008; Zaja-Milatovic *et al*, 2005) and D2 receptor gene expression (Gerfen *et al*, 1990). Future studies pairing PET imaging and microscopy in animal models may best resolve questions regarding the contribution of the density of dopaminergic inputs to striatum *vs* their activity (eg individual differences in firing rate) in generating positive relationships between dopamine synthesis capacity and D2/3 receptor binding.

We did not find evidence of a relationship between striatal dopamine synthesis capacity and dopamine release measured with methylphenidate-paired [^11^C]raclopride. Within-subject relationships between dopamine synthesis and release have not been previously reported. However, one study found both elevated dopamine synthesis capacity and elevated dopamine release in two independent groups of immigrants relative to non-immigrant controls (Egerton *et al*, 2017). There are many factors that contribute to the rate and volume of neurotransmitter release for which regulation may be independent of aromatic amino acid decarboxylase activity. These factors may have contributed to the lack of correlation between [^18^F]FMT K_i_ and change in [^11^C]raclopride BP_ND_ following methylphenidate. These may include, but are not limited to, the activity of the vesicular monoamine transporter, the activity of vesicular tracking proteins, and the distribution of vesicles in the readily releasable pool. In rodent models, ^11^C labeling of dopamine precursors suggest that newly synthesized dopamine is not detectable in the synapse, but is stored in synaptic vesicles not immediately released (Okada *et al*, 2011). These findings suggest some degree of uncoupling between synthesis and release, at least in the time domain.

The methylphenidate-paired [^11^C]raclopride method for measuring dopamine release may be complicated by individual differences in the effect of methylphenidate on dopamine transporters. Volkow *et al* (1998a, 2002) have used [^11^C]cocaine PET to estimate dopamine transporter binding and displacement after methylphenidate administration. Though there are individual differences in [^11^C]cocaine displacement with methylphenidate, decreases in transporter binding were not correlated with decreases in [^11^C]raclopride binding with methylphenidate (Volkow *et al*, 2002). The authors concluded that variability in methylphenidate binding to the transporter was not the primary source of individual differences in changes in [^11^C]raclopride BP_ND_ with methylphenidate. This, however, does not rule out the possibility that differences in transporter function contribute to estimated dopamine release (see discussion in Volkow *et al*, 2001). Indeed, there was modest indication of a relationship between baseline [^11^C]cocaine and release measures (*r*=0.34), though sample size was limited (*n*=10; Volkow *et al*, 2002).

It is possible that relationships between dopamine synthesis capacity and dopamine release can be unmasked with alternative pharmacological treatments. The 30 mg methylphenidate pill used here produced average change in [^11^C]raclopride BP_ND_ of 8.78%. This is within the 5.3–10.2% change range described for [^11^C]raclopride BP_ND_ observed during cognitive task performance (Jonasson *et al*, 2014; Monchi *et al*, 2006). Higher 60 mg oral methylphenidate amounts produce [^11^C]raclopride BP_ND_ reductions of approximately 11–20% (Volkow *et al*, 2001; Volkow *et al*, 2002; Martinez *et al*, 2011; Martinez *et al*, 2012; Broft *et al*, 2012). To further probe the null result observed here, future studies should test whether using higher methylphenidate doses or, alternatively, using amphetamine to stimulate dopamine release (Kuczenski and Segal, 1989; Sharp *et al*, 1987) reveals relationships between [^18^F]FMT K_i_ and changes in [^11^C]raclopride BP_ND_. Additionally, future studies would be strengthened by the use of plasma testing of d-threo methylphenidate (Volkow *et al*, 1998b) and fixed mg/kg doses, the absence of which represent limitations in the present study.

This study sheds light on the unique information conveyed by pre- and post- synaptic measures of dopaminergic function in healthy adults. Though there were positive relationships between [^18^F]FMT and baseline [^11^C]raclopride measures, the strength of these correlations were relatively weak (*r*=0.26–0.46) indicating [^18^F]FMT and [^11^C]raclopride cannot simply be used as proxy measures for one another in healthy populations. Considering the limitations of the present study, it is also possible that further screening and stricter exclusion of participants would have strengthened the observed relationships. Specifically, we did not submit participants to urine drug screens on the day of PET scanning, did not include the Structured Clinical Interview for DSM Disorders, and did not exclude for family history of psychiatric disorder, which may affect dopamine synthesis capacity (Huttunen *et al*, 2008).

This study establishes relationships between pre- and postsynaptic dopamine function in healthy young adults that can be tested in other populations. Alterations in dopamine function are associated with aging, and are a central component of disorders including Parkinson’s disease, schizophrenia, and addiction. Evidence for compensatory regulation of dopamine function may be most clear in such populations, where the positive relationships between estimated dopamine synthesis and receptor density observed in healthy adults may disappear with disease or show a reversal in their relationship. For example, in aging, different studies report decreased density of D2/3 receptors (Backman *et al*, 2000; Kuwabara *et al*, 2012; Volkow *et al*, 1998a; Volkow *et al*, 1996) accompanied by increases in dopamine synthesis capacity (Berry *et al*, 2016; Braskie *et al*, 2008).

In summary, our study revealed positive relationships between presynaptic dopamine synthesis capacity and postsynaptic D2/3 receptor binding measures, but failed to provide evidence supporting our hypothesis that dopamine synthesis and release would be positively related. Our results underscore the importance of empirical testing of the interrelationships between dopamine measures, and take initial steps in defining the balance of multiple aspects of the dopamine system in healthy adults. Our findings may offer a template from which to characterize alteration in striatal dopamine function in disease.

## Funding and disclosure

This research was generously funded by NIH grants R01 DA034685, R01 AG044292, F32 AG047686, and F32 DA038927. The authors declare no conflict of interest.

## Figures and Tables

**Table 1:** PET Signal in Striatal Regions of Interest

	[^**18**^**F]FMT K**_**i**_	[^**11**^**C]RAC BP**_**ND**_	[^**11**^**C]RAC BP**_**ND**_	[^**11**^**C]RAC BP**_**ND**_
		**placebo**	**methylphenidate**	**% change**
whole striatum	0.015±0.002	2.19±0.18	1.99±0.18	8.78±4.23
dorsal caudate	0.019±0.002	3.02±0.27	2.79±0.27	7.48±3.78
dorsal putamen	0.024±0.002	3.85±0.27	3.48±0.33	9.63±5.88
ventral striatum	0.018±0.002	2.61±0.22	2.40±0.24	8.12±4.13

Values reflect mean±standard deviation. % change was calculated as 100 x ([^11^C]raclopride placebo-[^11^C]raclopride methylphenidate)/ [^11^C]raclopride placebo. [^11^C]raclopride is abbreviated as [^11^C]RAC. degrees of freedom=39.

**Table 2:** Correlations between PET measures in striatal regions of interest

	[^**18**^**F]FMT** ***vs***	[^**11**^**C]RAC placebo** ***vs***	[^**18**^**F]FMT** ***vs***
	[^**11**^**C]RAC placebo**	[^**11**^**C]RAC methylphenidate**	[^**11**^**C]RAC (% change)**
whole striatum	*r*=0.46 (0.17, 0.70), *p*=0.003	*r*=0.86 (0.76, 0.92), *p*<0.001	*r*=−0.01 (−0.31, 0.33), *p*=0.974
dorsal caudate	*r*=0.34 (0.07, 0.57), *p*=0.031	*r*=0.90 (0.81, 0.95), *p*<0.001^a^	*r*=−0.15 (−0.43, 0.15), *p*=0.352
dorsal putamen	*r*=0.26 (−0.05, 0.52), *p*=0.102	*r*=0.72 (0.58, 0.82), *p*<0.001^a^	*r*=0.01 (−0.26, 0.28), *p*=0.936
ventral striatum	*r*=0.41 (0.18, 0.63), *p*=0.008^a^	*r*=0.90 (0.83, 0.94), *p*<0.001^a^	*r*=0.11 (−0.25, 0.44), *p*=0.522

R-values (95% confidence interval) and *p*-values are reported. Correlations between [^18^F]FMT and [^11^C]raclopride % change: (placebo – methylphenidate)/placebo) are corrected for individual differences in body weight. [^11^C]raclopride is abbreviated as [^11^C]RAC. For striatal subregions.

a Indicates relationships surviving Bonferroni correction for three comparisons.
