# Supplementary material for: Dopamine Synthesis Capacity is Associated with D2/3 Receptor Binding but Not Dopamine Release
Source: Neuropsychopharmacology. 2017 Oct 4;43(6):1201–11. doi: 10.1038/npp.2017.180 (PMC5916345; doi:10.1038/npp.2017.180)
Supplement: Supplementary Tables [file npp2017180x1.docx]

Table S1: Partial volume corrected PET signal in manually drawn striatal regions of interest. Values reflect mean ± standard deviation. % change was calculated as 100 x ([^11^C]raclopride placebo-[^11^C]raclopride methylphenidate)/ [^11^C]raclopride placebo. [^11^C]raclopride is abbreviated as [^11^C]RAC. degrees of freedom = 39.

|  | [^18^F]FMT K_i_ |  | [^11^C]RAC BP_ND_ | [^11^C]RAC BP_ND_ | [^11^C]RAC BP_ND_ |
| --- | --- | --- | --- | --- | --- |
|  |  |  | placebo | methylphenidate | % change |
| dorsal caudate | 0.026 ± 0.003 |  | 6.17 ± 0.76 | 5.76 ± 0.76 | 6.72 ± 5.95 |
| dorsal putamen | 0.032 ± 0.004 |  | 7.24 ± 0.72 | 6.55 ± 0.76 | 9.38 ± 6.79 |
| ventral striatum | 0.021 ± 0.004 |  | 4.03 ± 0.52 | 3.70 ± 0.57 | 8.37± 7.06 |

Table S2: Correlations between partial volume corrected PET measures in manually drawn striatal regions of interest. R-values [95% confidence interval] and p-values are reported. Correlations between [^18^F]FMT and [^11^C]raclopride % change (([^11^C]raclopride placebo-[^11^C]raclopride methylphenidate)/ [^11^C]raclopride placebo) are corrected for individual differences in body weight. [^11^C]raclopride is abbreviated as [^11^C]RAC. * indicates relationships surviving Bonferroni correction for 3 comparisons.

|  | [^18^F]FMT vs | [^11^C]RAC placebo vs | [^18^F]FMT vs |
| --- | --- | --- | --- |
|  | [^11^C]RAC placebo | [^11^C]RAC methylphenidate | [^11^C]RAC (% change) |
| dorsal caudate | r = .22 [-.06, .44], p = .168 | r =.90 [.81, .95] , p < .001* | r = -.21 [-.51, .15], p = .191 |
| dorsal putamen | r = .46 [.13, .69], p =.003* | r = .76 [.61, .87], p < .001* | r = .15 [-.17, .45], p = .343 |
| ventral striatum | r = .47 [.13, .68], p = .002* | r = .86 [.72, .95], p < .001* | r = .13 [-.18, .48], p = .418 |
